# A Comprehensive Screening and Identification of Genistin Metabolites in Rats Based on Multiple Metabolite Templates Combined with UHPLC-HRMS Analysis

**DOI:** 10.3390/molecules23081862

**Published:** 2018-07-26

**Authors:** Yaoyue Liang, Wenjing Zhao, Chenxiao Wang, Zijian Wang, Zhibin Wang, Jiayu Zhang

**Affiliations:** 1 School of Chinese Pharmacy, Beijing University of Chinese Medicine, Beijing 100029, China; lyy2610@163.com (Y.L.); cosyzwj@126.com (W.Z.); 18801381993@163.com (C.W.); 2 Beijing Research Institution of Chinese Medicine, Beijing University of Chinese Medicine, Beijing 100029, China; helloffiresilver@gmail.com

**Keywords:** genistin, UHPLC-LTQ-Orbitrap mass spectrometer, multiple metabolite templates, metabolic profiling

## Abstract

Genistin, an isoflavone belonging to the phytoestrogen family, has been reported to possess various therapeutic effects. In the present study, the genistin metabolites in rats were investigated by UHPLC-LTQ-Orbitrap mass spectrometer in both positive and negative ion modes. Firstly, the data sets were obtained based on data-dependent acquisition method and then 10 metabolite templates were established based on the previous reports. Then diagnostic product ions (DPIs) and neutral loss fragments (NLFs) were proposed to efficiently screen and ascertain the major-to-trace genistin metabolites. Meanwhile, the calculated Clog *P* values were used to identify the positional isomers with different retention times. Consequently, a total of 64 metabolites, including prototype drug, were positively or putatively characterized. Among them, 40 metabolites were found according to the templates of genistin and genistein, which was the same as the previous research method. After using other metabolite templates, 24 metabolites were added. The results demonstrated that genistin mainly underwent methylation, hydrogenation, hydroxylation, glucosylation, glucuronidation, sulfonation, acetylation, ring-cleavage and their composite reactions *in vivo* biotransformation. In conclusion, the research not only revealed the genistein metabolites and metabolic pathways *in vivo* comprehensively, but also proposed a method based on multiple metabolite templates to screen and identify metabolites of other natural compounds.

## 1. Introduction

Recently, isoflavones have received much attention due to their nutritional and potential health benefits. They are non-steroidal phytoestrogenic and antioxidative polyphenolic molecules, which have the possibilities to protect against hormone-dependent diseases, such as prostate cancer, breast cancer, menopausal symptoms, cardiovascular disease and osteoporosis [1,2,3]. Genistin, as one of the common isoflavones, has a similar structure with phytoestrogens and belongs to the annual plant of Fabaceae family especially *Glycine max* (L.) MERR. It possesses various therapeutic effects, including anti-inflammatory, anticancer activities, cardioprotective effects, antioxygenation, *etc.*, through pharmacological studies [4,5,6,7]. For example, it was found that genistin could enhance the expression of acetylcholinesterase (AChE) related enzymes and related proteins in cultured rat osteoblasts. Therefore, it was speculated that genistin could improve osteoporosis [8]. Compared with other drugs, genistin has many advantages, such as abundant sources, stable nature, low toxicity and side effects. Although there is no clear evidence that the ingestion of genistin is harmful, genistin (*i.e.*, genistein-7-glucoside) can metabolize to produce genistein after entering the body. The genotoxicity and potential adverse effects (cell growth inhibition, apoptosis, topoisomerase inhibition, and DNA damages) of genistein were reported *in vitro* as well as in experimental animals (*e.g.*, rats) [9,10,11,12,13]. Genistin produces metabolic products of different biological effects through metabolic process *in vivo*, and the metabolic level of different organisms is different. However, it is still ambiguous that what the material basis is responsible for the effects of genistin. But it is certain that metabolites play an important role. Therefore, it is necessary to study the metabolism of genistin, which helps to explore its effects on cardiovascular and cerebrovascular diseases, tumor and other diseases. And it is of great significance to understand the biological activity of isoflavones and explore its development and utilization.

Contemporarily, ultra-high performance liquid chromatography (UHPLC) coupled with mass spectrometry (MS) has been widely used in drug metabolism analysis [14,15,16]. UHPLC has a powerful separation capability, which is currently one of the most effective tools for the complex components separation. MS owns the properties of high speed, sensitivity and selectivity, during which the high resolution (HR) and multistage mass spectrometry technology have been comprehensively used for online structural analysis and quantitative detection of known and unknown components. In recent years, with the continuous development of Orbitrap technology, Orbitrap mass spectrometer is widely used in pharmaceutical analysis due to the HR detection through combining with the linear ion trap [17,18,19]. Nowadays, UHPLC-LTQ-Orbitrap mass spectrometer has been widely applied in metabolism studies [20,21,22,23,24].

To date, there are dozens of reports concerned with the techniques of dealing the dataset to extract the metabolite information. These techniques include the adoption of mass defect filters, isotope pattern filters, and background subtraction and so on [25]. Generally, the parent drug is selected as the template to predict metabolites according to the common metabolic pathways. The obvious limitation is that many drug metabolites are yielded through the composite reactions. In the previous studies of the metabolites of genistin and the corresponding aglycone (genistein), the number of the detected metabolites was small [26,27,28,29]. This greatly hinders the discussion of the material basis for the efficacy of genistin after entering the body. The characteristic of our study is that according to the literature reports and the results of pre experiments, the multiple metabolite templates are established to assist with data processing. In combination of diagnostic product ions (DPIs), neutral loss fragments (NLFs) and calculated Clog *P* values, the potential metabolites of genistin were proposed in this study. As far as we know, it was the first time to comprehensively investigate the metabolism of genistin *in vivo*.

## 2. Results and Discussion

### 2.1. The Establishment of Analytical Strategy

**Figure 1:**
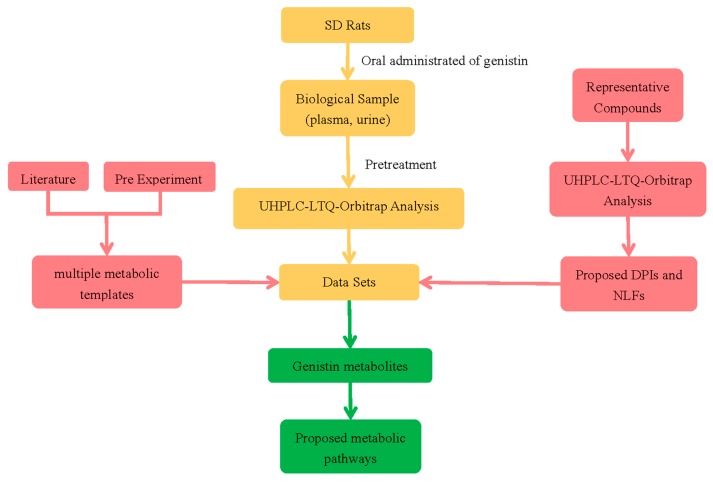
The summary diagram of analytical strategy for detection and identification of genistin metabolites.

In this study, we established a comprehensive and effective strategy to discover and identify genistin metabolites by using UHPLC-HRMS. At the beginning, HR-ESI-MS^1^ analysis was performed on the HRMS instrument with a resolution of 30,000. Meanwhile ESI-MS^n^ data sets were got both in negative and positive ion modes based on data-dependent acquisition method. Secondly, the metabolite templates were established according to the literature reports and the pre experiment results. Then DPIs and NLFs, which were proposed by the mass fragmentation behaviors of reference standards, were applied to efficiently confirm and identify the genistin metabolites. After that, according to different retention times, positional isomers were distinguished by using the corresponding Clog *P* values calculated by ChemBioDraw Ultra 14.0 (PerkinElmer, Waltham, MA, USA). Depending on the information obtained above, all metabolites were identified positively or putatively. Finally, grounded on metabolites data and references, the metabolic pathway of genistin was proposed. Through the whole experiment, we had summed up a schematic diagram, as shown in Figure 1, which was an analytical strategy for detecting and identifying genistin metabolites.

### 2.2. The Establishment of Multiple Metabolite Templates Screening Method

Ten metabolite templates, genistin, genistein, daidzin, daidzein, puerarin, dihydrogenistein, tetrahydrogenistein, dihydrodaidzein, equol, and *O*-desmethylangolensin, were selected as the screening templates for genistin metabolites. Of course, based on this method, the metabolites selected from different templates would be the same. Combined with the retention time, DPIs, and NLFs, the compounds with the same structure need to be deleted. Based on this efficient method, some uncommon compound metabolites could be much more comprehensively detected.

### 2.3. Establishment of Diagnostic Product Ions Basing on Fragmentation Behaviors of Genistin and Its Homologues

**Figure 2:**
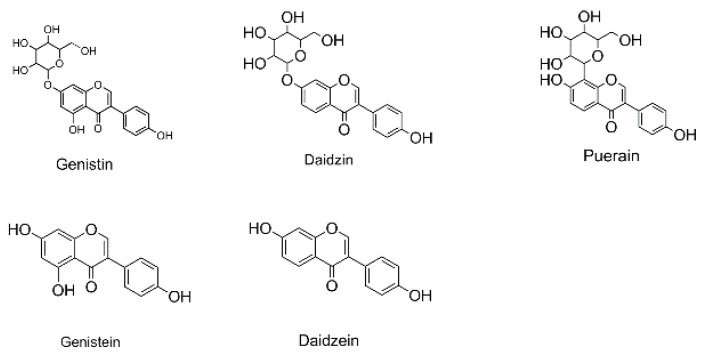
The chemical structures of the five reference standards.

**Figure 3:**
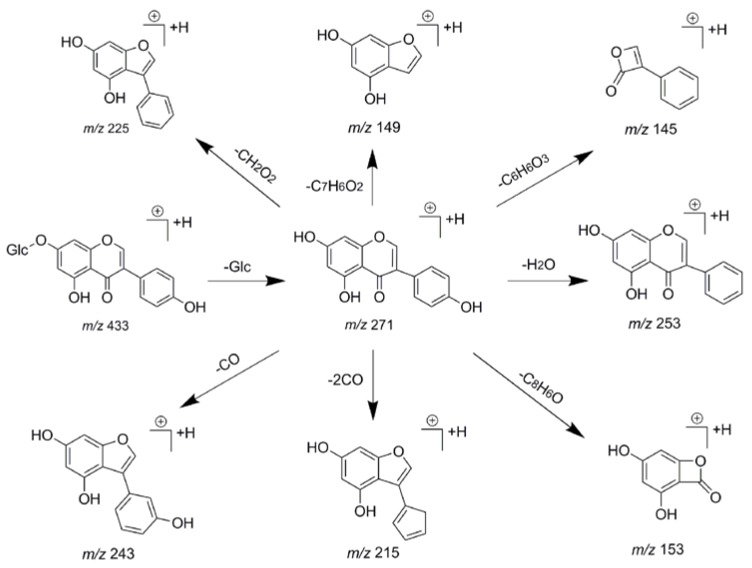
The fragmentation behavior of genistin in positive ion mode.

In order to obtain an extensive fragmentation behavior of genistin and the other four homologues (genistein, daidzin, daidzein, and puerarin) (Figure 2), the mixed standard solution was comprehensively analyzed by using UHPLC-LTQ-Orbitrap MS. It can provide the useful information, such as DPIs and characteristic fragmentation patterns, which were utilized for deducting the structures of related metabolites. Taking genistin as an example, it showed a protonated [M + H]^+^ ion at *m*/*z* 433.1110 (C_21_H_21_O_10_, −4.39 ppm) with the retention time of 4.59 min in the ESI-MS experiment. It produced the base peak ion at *m*/*z* 271 [M + H − 162]^+^ via the loss of glucose moiety in the ESI-MS^2^ spectrum. In the ESI-MS^3^ experiment, *m*/*z* 153 was yielded as the base peak via Retro-Diels-Alder (RDA) fragmentation by losing C_8_H_6_O. Meanwhile, the other characteristic ions at *m*/*z* 215 (81%), 243 (59%), 253 (34%), 149 (30%), 145 (16%), and 225 (11%) were yielded by losing 2CO, CO, H_2_O, C_7_H_6_O_2_, C_6_H_6_O_3_, and CO + H_2_O, as shown in Figure 3. For genistein with retention time of 8.00 min, the MS^2^ ions at *m*/*z* 153 (100%), 215 (79%), 243 (62%), 253 (34%), 149 (29%), 145 (18%), and 225 (10%) were the same as the ESI-MS^3^ ions of genistin, which could be attributed to the fact that genistin is the glucoside of genistein. Meanwhile, daidzin and daidzein were eluted at 3.86 and 6.28 min, respectively. In the MS^3^ spectrum of daidzin and MS^2^ spectrum of daidzein, the most abundant fragment ion at *m*/*z* 199 was produced due to the loss of 2CO. Moreover, several major fragment ions at *m*/*z* 137, *m*/*z* 227, *m*/*z* 237, and *m*/*z* 145 were also observed due to the respective loss of C_8_H_6_O, CO, H_2_O, and C_6_H_6_O_2_, which indicated that they owned the similar cracking behavior with genistin [26].

**Figure 4:**
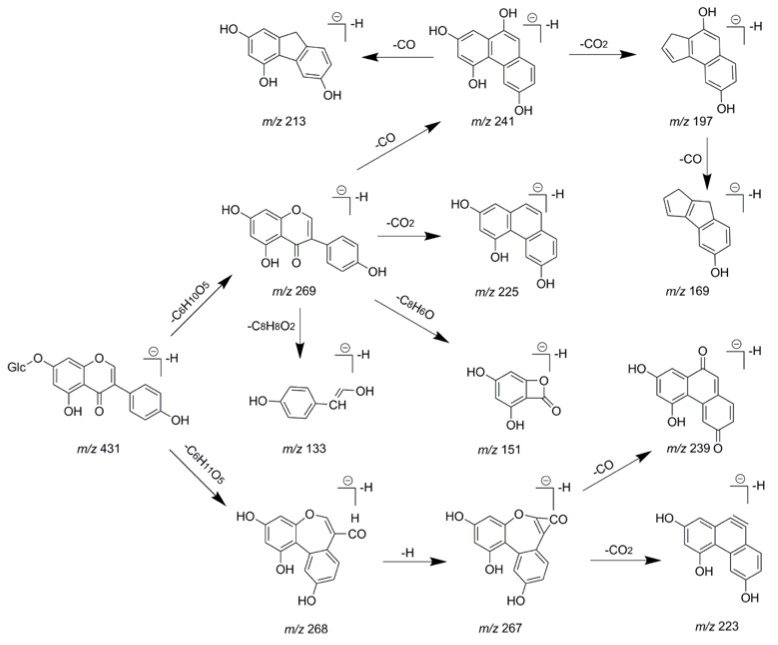
The fragment behaviors of genistin in negative ion mode.

In addition, it was necessary to note that *m*/*z* 269 and *m*/*z* 268 were produced in the ESI-MS^2^ of genistin in negative ion mode under the lower collision energies. It showed the loss of a C_6_H_10_O_5_ (162 Da) moiety and the corresponding pure scission process by losing C_6_H_11_O_5_ (163 Da) moiety, which resulted in the formation of the aglycone and free radical aglycone product ions [30]. Some of the main characteristic ions were generated by rearrangement and scission. For example, *m*/*z* 267 (100%), 239 (81%), and 223 (56%) were occurred in the ESI-MS^3^ spectrum. The residues in the ESI-MS^3^ of genistin were similar to those in the ESI-MS^2^ spectrum of genistein as showed in Figure 4, which could facilitate the structural elucidation of the genistin metabolites *in vivo*. Besides, the fragmentation pathways of daidzin and daidzein were also similar to those of genistin [31].

**Figure 5:**
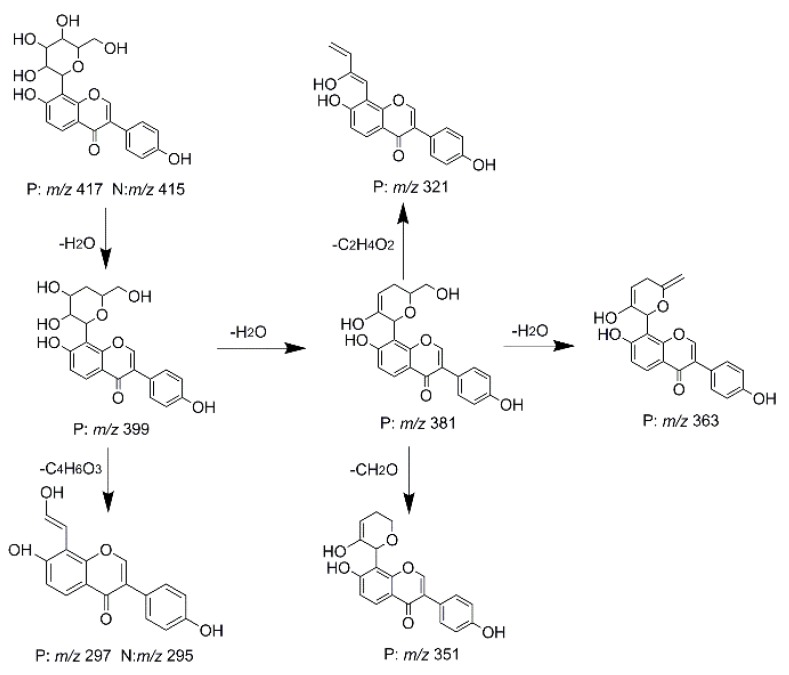
The fragmentation patterns of puerarin (P for the positive ion mode and N for the negative ion mode).

Unlike the above four reference standards, puerarin, attributed to C-glycoside, possessed its own unique cleavage mode. Taking puerarin in positive ion mode as an example, it yielded the protonated molecule [M + H]^+^ ion at *m*/*z* 417.1160 (C_21_H_21_O_9_, −4.89 ppm) in the ESI-MS^1^ spectrum. In the ESI-MS^2^ spectrum, some fragmentation ions, occurred on heteroside moiety, were generated, including *m*/*z* 399 (100%), *m*/*z* 351 (53%), *m*/*z* 381 (36%), *m*/*z* 297 (31%), *m*/*z* 363 (14%), and *m*/*z* 321 (12%) by NLFs of H_2_O, 2H_2_O + CH_2_O, 2H_2_O, C_4_H_6_O_3_, 3H_2_O, and 2H_2_O + 2CH_2_O. The fragmentation pathways of puerarin in positive and negative ion modes are proposed in Figure 5 [32,33].

To facilitate the structural illustration of genistin metabolites in the complex matrix, the DPIs were applied to distinguish genistin metabolites in this study [25]. The homologues possessed the similar fragmentation behaviors, which meant that there would be similar DPIs and regular NLFs for providing adequate evidences for elucidating the metabolites. With the biological reactions *in vivo*, the DPIs might change accordingly. For instance, in positive ion mode, the aglycone moiety of genistin was easily lost in ESI-MS analysis, and the DPI at *m*/*z* 271 [M + H − C_6_H_10_O_5_]^+^ would be produced in ESI-MS^2^ spectrum. If some different kinds of biological reactions occurred on the original drug (not on the aglycone moiety), new DPIs resulted from newly generated metabolites at *m*/*z* 271 + X (X: mass weight of substituent groups, such as 14, 16, 80, 162, *etc.*) giving information about the type of the substituent groups. Besides, the DPI at *m*/*z* 153 was generated due to RDA rearrangement which occurred on the 1,4-position of C-ring of genistin in positive ion mode. It would give the information whether bio-reactions occurred on the A-ring or not. To point out, NLFs also provided tremendous help for efficiently elucidating the structures of unknown metabolites. For instance, the successive losses of 28 Da (CO) eliminated in the ESI-MS^n^ spectra of genistin and the NLF of 44 Da (CO_2_) was only found in negative ion mode of genistin, which could also offer much information to identify these metabolites. Although the metabolites were difficult to identify because of the absence of corresponding reference standards, the DPIs coupled with NLFs were calculated to clarify these complex metabolites.

### 2.4. Identification of Genistin Metabolites in Rats

**Figure 6:**
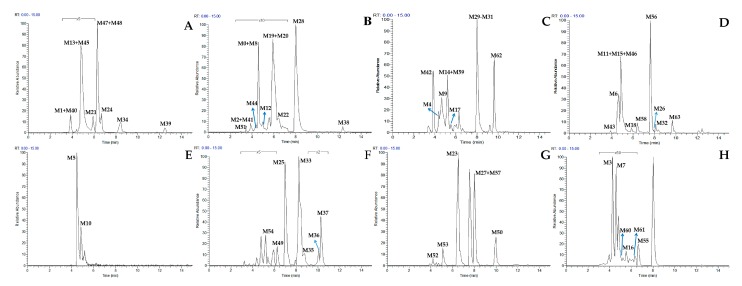
High resolution extracted ion chromatograms of genistin metabolites ((**A**–**E**) for negative ion mode and (**F**–**H**) for positive ion mode): (**A**) *m*/*z* 253.0495, 283.0600, 299.0549, 447.0922; (**B**) *m*/*z* 269.0444, 349.0012, 415.1023, 431.0972; (**C**) *m*/*z* 225.0546, 227.0340, 241.0495, 257.0809, 364.9961, 417.1181, 429.0815, 447.0922, 473.1079, 593.1501; (**D**) *m*/*z* 201.0570, 255.0652, 271.0601, 285.0393, 287.0913, 333.0063, 445.0765, 461.1078; (**E**) *m*/*z* 447.0922; (**F**) *m*/*z* 227.0703, 299.0903, 301.0706, 303.0863, 315.0864; (**G**) *m*/*z* 215.0702, 269.0803, 273.0758, 287.0550, 287.0915; (**H**) *m*/*z* 243.1016, 271.0601, 275.0908, 285.0757, 287.0915.

The total ion chromatograms (TICs) of urine and plasma samples from the rats after oral administration of genistin were obtained by using UHPLC-LTQ-Orbitrap mass spectrometry. After comparing the drug-obtained samples with the corresponding control ones, 64 metabolites were detected in both positive and negative ion modes, among which 39 metabolites in positive ion mode and 43 metabolites in negative ion mode were identified in urine, while six metabolites in positive ion mode and eight metabolites in negative ion mode were identified in plasma. The high-resolution extracted ion chromatograms (HREICs) of related metabolites were showed in Figure 6 and the chromatographic retention times and some other concerned data were summarized in Table 1.

#### 2.4.1. Identification of Metabolites with the Templates of Genistin and Genistein

Based on the templates of genistin and its aglycone, genistein, 40 compounds were identified, labeled from **M0** to **M39**.

**M0** and **M12**, which possessed the same deprotonated [M − H]^−^ ions at *m*/*z* 431.0972 (C_21_H_19_O_10_, error within ±3.50 ppm), were eluted at 4.64 and 5.01 min, respectively. By comparing the full-scan MS/MS^n^ spectra and retention time with the reference standard, **M0** was unequivocally deduced to be the parent drug. The accurate mass weight and major product ions (*m*/*z* 268, *m*/*z* 269, *m*/*z* 223, *m*/*z* 224, and *m*/*z* 267, *etc.*) of **M12** were coincident with those of **M0**, indicating that it could be deduced as a genistin isomer.

**M4**, eluted at 4.38 min, gave rise to its [M + H]^+^ ion at *m*/*z* 595.1645 (C_27_H_31_O_15_, −2.11 ppm). It was 162 Da higher than that of genistin, indicating that it might be glucosylation product of genistin. In its ESI-MS^2^ spectrum, successive NLFs of 162 Da (*m*/*z* 595 → *m*/*z* 433 → *m*/*z* 271) were observed, which demonstrated that the glucosyl group was probably introduced into the glucose moiety.

**M17**, eluted at 5.52 min, possessed the experimental [M − H]^−^ ion at *m*/*z* 473.1092 (C_23_H_21_O_11_, 2.80 ppm), which was 42 Da higher than that of genistin, meaning that it might be an acetylation product of genistin. The ion at *m*/*z* 311 was observed by the NLF of 162 Da and the ion at *m*/*z* 269 was also found by successive losing the NLFs of 162 Da and 42 Da, which further confirmed our deduction.

Metabolites **M8**, **M16**, **M20**, **M28**, and **M38** showed their retention times at 4.61, 5.52, 5.92, 8.03, and 12.34 min, respectively, were exhibited the same theoretical [M + H]^+^ ion at *m*/*z* 271.0601 (C_15_H_11_O_5_, error within ±4.00 ppm) or the same theoretical [M − H]^−^ ion at *m*/*z* 269.0444 (C_15_H_9_O_5_, error within ±4.00 ppm). Among them, **M28** was determined as genistein by comparing the retention time and fragmentation behaviors with genistein reference standard. **M8**, **M16**, **M20**, and **M38** possessed the similar characteristic fragments (such as *m*/*z* 153, *m*/*z* 215, *m*/*z* 243, *m*/*z* 253, and *m*/*z* 225 in positive ion mode and *m*/*z* 181, *m*/*z* 201, *m*/*z* 225, and *m*/*z* 197 in negative ion mode) with **M28** and therefore they were preliminarily identified as the isomers of genistein under the present situation.

Metabolites **M1**, **M3**, **M7**, **M13**, **M24**, and **M39** with a protonated [M + H]^+^ ion at *m*/*z* 285.0757 (C_16_H_13_O_5_, error within ±3.50 ppm) or a deprotonated [M − H]^−^ ion at *m*/*z* 283.0600 (C_16_H_11_O_5_, error within ±3.50 ppm), were individually eluted at 3.93, 4.27, 4.59, 5.02, 6.62, and 12.44 min. They were 14 Da more than genistein whether in positive ion mode or in negative ion mode. The DPI at *m*/*z* 270 [M + H − CH_3_]^+^ indicated that **M1**, **M24**, and **M39** might be methylated products of genistein. The DPI at *m*/*z* 242 generated by the NLF of CO and the DPI at *m*/*z* 152 yielded by RDA rearrangement occurred on positions 1 and 3 further confirmed our conjecture. Due to a compound with a larger Clog *P* value would have a longer retention time in a reverse phase (RP) chromatographic system [34]. Therefore **M1**, **M24**, and **M39** were putatively thought as 5-*O*-methylgenistein (Clog *P*, 2.09), 4′-*O*-methylgenistein (Clog *P*, 2.99), and 7-*O*-methyl-genistein (Clog *P*, 2.99).

There was a NLF of 15 Da (CH_3_) from **M13**, indicating that a methyl group existed in **M13**. The subsequent MS^3^ of *m*/*z* 268 generated the fragment ion at *m*/*z* 240 by the NLF of 28 Da. According the limited information, **M13** was guessed as the *O*-methyl product of an isomer of genistein. Different from the above methylated products, the ion of losing 15 Da was not found in the mass spectrum of **M3** and **M7**. As a result, we considered they might be the C-methylgenistein. The DPI at *m*/*z* 109 yielded by RDA rearrangement occurred on positions 10 and 4 further confirmed our conjecture.

**M36** and **M37**, possessed experimental protonated molecular ions [M + H]^+^ at *m*/*z* 299.0906 (C_17_H_15_O_5_, −2.84 ppm) and *m*/*z* 299.0905 (C_17_H_15_O_5_, −3.08 ppm), were detected at 10.07 and 10.28 min, respectively. They were 14 Da higher than that of methylgenistein, so they were could be the dimethylation products of genistein. The sequential NLFs of 15 Da (*m*/*z* 299 → *m*/*z* 284 → *m*/*z* 269) occurred in ESI-MS^n^ spectra, coupling with the DPIs at *m*/*z* 152 and *m*/*z* 166 (generated by the RDA rearrangement occurred on positions 1 and 3) further validated that one methyl group was added to A-ring, while the other methyl was introduced to B-ring. They were tentatively interpreted as 5,4′-dimethygenistein and 7,4′-dimethygenistein.

**M6**, **M11**, and **M15** with their retention times of 4.56, 4.88, and 5.11 min separately, were 176 Da higher than that of genistein. They exhibited the same molecular ion at *m*/*z* 445.0765 ([C_21_H_17_O_11_]^−^, error within ±3.50 ppm) and showed the NLF of 176 Da (*m*/*z* 445 → *m*/*z* 269) in their ESI-MS^2^ spectra, which indicated the presence of glucuronidation group. Meanwhile, the mass fragmentation behaviors of *m*/*z* 269 were similar to those of genistein, which provided adequate evidence that they were genistein-*O*-glucuronides. Due to the structure of genistein, there were three sites that might accomodate glucose aldehyde acidification. So **M6**, **M11**, and **M15** were provisionally characterized as 5-*O*-glucuronide-genistein, 7-*O*-glucuronide-genistein, and 4′-*O*-glucuronide-genistein based on their Clog *P* values.

Metabolites **M2**, **M19**, and **M22**, whose elution times were 3.96, 5.83, and 6.38 min, were 80 Da higher than that of genistein in negative ion mode, which indicated the occurrence of sulfonation reaction. In its MS/MS^2^ spectrum, the DPI at *m*/*z* 269 was detected due to the NLF of 80 Da from the parent ion at *m*/*z* 349 and in its MS/MS^3^ spectrum, the similar characteristic fragments with genistein were observed, which all indicated that the three metabolites were the sulfonation products of genistein. According to their Clog *P* values, they were briefly inferred to be 5-*O*-sulfate-genistein, 4′-*O*-sulfate-genistein, and 7-*O*-sulfate-genistein, respectively.

Metabolites **M18** and **M23** were eluted at 5.82 and 6.53 min, respectively, and produced the same theoretical [M − H]^−^ ion at *m*/*z* 285.0393 (C_15_H_9_O_6_, error within ±2.50 ppm) in negative ion mode, which were 16 Da higher than that of genistein. Thus they were supposed to be hydroxylation products of genistein due to the similar fragmentation behaviors. The DPI at *m*/*z* 153 (RDA rearrangement occurred on positions 1 and 3 of C-ring) of **M23** in positive ion mode, indicating that the hydroxyl group was added to the B-ring, which was deduced as 3′-hydroxygenistein [35]. **M18** was provisionally characterized as 8-hydroxygenistein on the basis of the calculated Clog *P* values [36]. **M9**, possessed the same fragment ions (*m*/*z* 285, 257, 229, and 217) with **M23** in negative ion mode, was 80 Da more than that of **M23**, manifesting the presence of sulfuric group.

**M5**, **M10**, and **M14**, whose retention times are 4.47, 4.85, and 5.11 min, were 162 Da more than that of hydroxylation of genistein, indicating they might be glucosylation products of hydroxylated genistein. The DPI at *m*/*z* 285 ([M − H − Glc]^−^) was yielded by a NLF of glucose moiety, which identified our hypothesis. Therefore, they were putatively identified as hydroxygenistein-*O*-glucoside.

**M21**, **M33**, **M34**, and **M35** were eluted at 5.92, 6.31, 8.24, 8.46, and 8.79 min, respectively. **M21** possessed a deprotonated molecule ion at *m*/*z* 299.0549 (C_16_H_11_O_6_, 2.89 ppm) and **M33**, **M34**, and **M35** showed a protonated molecule ion at *m*/*z* 301.0706 (C_16_H_13_O_6_, error within ±2.50 ppm). The NLF of 30 Da (CH_3_O) was observed in the ESI-MS^2^ spectra of **M21**, which indicated that it was a product of *O*-methoxygenistein. In the ESI-MS^3^ spectra, the similar characteristic ions were tested with genistein, further confirming our speculation. The DPIs of **M33**, **M34**, and **M35** were *m*/*z* 286 by loss of 15 Da (CH_3_) and *m*/*z* 258 via a NLF of 28 Da (CO), suggesting that they were the methylation of hydroxylated genistein.

The ion at *m*/*z* 153 of **M34** and **M35** were produced by RDA rearrangement occurred on positions 1 and 3 of C-ring, suggesting that hydroxylation and methylation were occurred on B-ring. Therefore they were identified as 3′-hydroxyl-4′-*O*-methylgenistein and 3′-methoxylgenistein, respectively. The fragment ion at *m*/*z* 168 of **M33** indicated that hydroxyl was attributed to A-ring.

**M25**, 14 Da more than that of **M34**, presented a protonated molecule ion at *m*/*z* 315.0856 (C_17_H_15_O_6_, −2.36 ppm) with the retention time of 6.98 min. The DPI at *m*/*z* 300 with the loss of CH_3_ and *m*/*z* 167 produced by the RDA rearrangement occurred on positions 1 and 3 provided the evidence for identifying the metabolite. In addition, the fragmentation behaviour was similar with **M34**. Hence we deemed **M25** was the methylated product of **M34** with the methyl group attaching to A-ring.

**M26**, **M27**, and **M29**-**M32** were the cleavage products of genistein by losing different groups. The metabolite **M27** with the quasi-molecular ion at *m*/*z* 215.0697 (C_13_H_11_O_3_, −2.65 ppm) was discovered at 7.97 min in the ESI-MS^1^ spectra. In the MS^2^, the base peak of *m*/*z* 197 was generated by loss of C_2_H_2_O_3_ (2CO + H_2_O). Based on the possible metabolic path, its structure was suspected as showed in Table 1. In negative ion mode, **M26** and **M32** showed the [M − H]^−^ at *m*/*z* 201.0553 (C_12_H_9_O_3_, 3.38 ppm) and 201.0552 (C_12_H_9_O_3_, 2.78 ppm), respectively. The characteristic fragment ions were *m*/*z* 173, 159, 157, and 133 by loss of CO, C_2_H_2_O, CO_2_, and C_4_H_4_O. According to the above data, both compounds were decided as showed in Table 1.

**M29** (C_14_H_9_O_3_) and **M30** (C_14_H_9_O_4_) were generated via the loss of CO_2_ and CO at ring C, yielding [M − H − CO_2_] (*m*/*z* 225.0546, −0.09 ppm) and [M − H − CO] (*m*/*z* 241.0494, −0.52 ppm), respectively. Both ions possessed stable structures with a basic phenanthrene framework. The same fragmentation behaviour with genistein in negative ion mode supported our speculation. Different from **M29** and **M30**, **M31** was formed by the loss of C_2_H_2_O occurred at ring A, which was confirmed by its further fragmentation behavior with losing CO_2_, CO, and CO + CO_2_. Therefore, the structures were showed in Table 1.

#### 2.4.2. Identification of Metabolites with the Templates of Daidzin and Daidzein

**M40**, **M45**, and **M48** gave a deprotonated ion at *m*/*z* 253.0495 (C_15_H_9_O_4_, error within ±4.00 ppm), which were eluted at 3.85, 4.82, and 6.33 min, respectively. By comparing the chromatographic retention time and MS/MS spectra, **M48** was positively identified as daidzein. Due to the similar fragmentation behaviours with **M48**, we conjectured **M40** and **M45** were the isomerides of daidzein.

**M41** and **M44** were 162 Da higher than daidzein, indicating that they were the glucoside conjugate of daidzein. In the MS^2^, one fragment ion, *m*/*z* 255, was formed by the loss of the glucose moiety from the precursor ion. In the MS^3^, the neutral losses of CO, 2CO, and C_8_H_6_O from *m*/*z* 255 were observed and the corresponding fragment ions at *m*/*z* 227, 199, and 137 were generated, respectively. Based on the reference compound, **M41** was hopefully identified as daidzin, therefore **M44** was deduced as 4′-glucoside-daidzein and the calculated Clog *P* value further confirmed our guess. **M43**, showed the [M − H]^−^ ion at *m*/*z* 461.1091 (C_22_H_21_O_11_, 2.80 ppm), was 46 Da more than daidzin and the highest product ion at *m*/*z* 253 was found in the mass spectrum of MS^2^. The second highest product ion at *m*/*z* 415 was formed via the loss of CH_2_O_2_. Hence we suspected the structure of **M43** as showed in Table 1.

**M42** exhibited a protonated ion at *m*/*z* 431.0973 (C_21_H_19_O_10_, −2.44 ppm), which was 176 Da higher than that of daidzein. MS^2^ gave the base peak of *m*/*z* 255 via loss of a glucuronide moiety (176 Da) from *m*/*z* 431. In the MS^3^ spectrum of **M42**, the *m*/*z* 255 ion yielded specific ions at *m*/*z* 199, 137, 227, and 145 due to the loss of 2CO, C_8_H_6_O, CO, and C_6_H_6_O_2_. Therefore **M42** was supposed as the daidzein-*O*-glucuronide. The MS^2^ of **M46** ([M + H]^+^, *m*/*z* 335.0208) generated the fragment ion at *m*/*z* 255 by the NLF of 80 Da and the fragmentation behaviour of *m*/*z* 255 was similar with daidzein by losing 2CO, C_8_H_6_O, CO, and C_6_H_6_O_2_, so we proposed that **M46** was *O*-sulfate-daidzein.

**M50**, eluted at 9.92 min, was 14 Da higher than daidzein in positive ion mode, manifesting that it might be a methylated product of daidzein. The DPIs at *m*/*z* 254 (100%), 237 (46%), and 213 (37%) were generated by the NLFs of CH_3_, CH_4_O (H_2_O + CH_2_), and 2CO. Because of two active sites existed in daidzein, **M50** was putatively appraised as *O*-methyldaidzein.

**M47** was eluted at 6.31 min with a deprotonated [M − H]^−^ ion at *m*/*z* 299.0549 (C_16_H_11_O_6_, −0.48 ppm). It was 46 Da higher than that of daidzein, showing that there was a CH_2_O_2_ more than daidzein in molecular composition. The DPIs at *m*/*z* 253, 225, and 224 offered the evidence to support our guess. The ion at *m*/*z* 163 was formed by RDA rearrangement which occurred on positions 0 and 3 on C-ring, which indicated that the CH_2_O was attached to B-ring as shown in Table 1 [36].

Metabolite **M49** with a protonated [M + H]^+^ ion at *m*/*z* 227.0698 (C_14_H_11_O_3_, −2.16 ppm), was eluted at 6.34 min. The DPI at *m*/*z* 199 was found in the mass spectrum of MS^2^ by the NLF of CO with no other characteristic ions observed. So it was putatively identified as the decarbonylation product of daidzein.

#### 2.4.3. Identification of Metabolites with the Templates of Puerarin

**M51**, possessed the deprotonated [M − H]^−^ ion at 415.1036 (C_21_H_19_O_9_, 2.94 ppm), was 162 Da more than that of daidzein with the retention time of 3.49 min. It was definitely identified as puerarin by comparing with the information of the standard.

#### 2.4.4. Identification of Metabolites with the Template of Hydrogenated Genistein

**M53**, **M56**, and **M57** were detected at 5.13, 7.64, and 8.03 min and possessed the same [M + H]^+^ ion at *m*/*z* 273.0758 (C_15_H_13_O_5_, error within ±3.00 ppm), which were 2 Da higher than that of genistein. In their ESI-MS^2^ spectra, DPIs of **M53** and **M56** at *m*/*z* 255 ([M + H − H_2_O]^+^) and *m*/*z* 245 ([M + H − CO]^+^) were found and *m*/*z* 153 of **M57** was also detected via RDA rearrangement occurred on positions 1 and 3. So we suspected that they were hydrogenated products of genistein and its isomers.

The two compounds of **M52** and **M55** showed [M + H]^+^ at *m*/*z* 287.0915 (C_16_H_15_O_5_, error within ±2.80 ppm), respectively, with 14 Da more than that of hydrogenated genistein. The quasi-molecular ion at *m*/*z* 287 presented the base peak at *m*/*z* 259 via the loss of 28 Da, while the base peak of **M55** at *m*/*z* 272 was produced via a loss of 15 Da, so we deduced that **M52** and **M55** were the C-methylated product and *O*-methylated product of genistein, respectively. **M54**, yielding the accurate [M + H]^+^ at *m*/*z* 303.0870, was 30 Da more than that of hydrogenated genistein. The product ions at *m*/*z* 285 (87%) and 257 (22%) were generated by the loss of H_2_O and H_2_O + CO. There was no other more information, so **M54** was putatively considered as a product of *C*-methyl-hydroxylation of genistein.

#### 2.4.5. Identification of Metabolites with the Template of Hydrogenated Daidzein

**M58** was 2 Da higher than that of daidzein, which indicated that it might be hydrogenation product of daidzein. The most abundant fragment at *m*/*z* 149 in the spectra of **M58** in negative ion mode was a result of RDA reaction occurred on the positions 2 and 3. The DPI at *m*/*z* 135 was generated by RDA rearrangement occurred between positions 1 and 3, further conforming our conjecture.

#### 2.4.6. Identification of Metabolites with the Template of Equol

**M60**, eluted at 5.23 min, gave rise to its [M + H]^+^ ion at *m*/*z* 243.1008 (C_15_H_15_O_3_, −3.05 ppm). The observed DPI of *m*/*z* 123 was generated by RDA rearrangement which occurred on positions 1 and 3 on C-ring. Therefore, **M60** was putatively identified as equol [37].

**M61** was 32 Da more than that of **M60**, indicating **M61** might be the dihydroxylated product of **M60**. In the ESI-MS^2^ spectra, the corresponding base peak at *m*/*z* 151 was generated by losing 124 Da (C_6_H_4_O_3_). The fragmentation ions of the subsequent MS^3^ of *m*/*z* 151 were similar with equol [37], which was consistent with our guess.

**M59**, possessing deprotonated [M − H]^−^ ion at 417.1194 (C_21_H_21_O_9_, 3.38 ppm), was eluted at 5.20 min, which was 176 Da higher than **M60**. Due to there was no other characteristic ions, **M59** was guessed as the product of glucose aldehyde acidification of equol.

#### 2.4.7. Identification of Metabolites with the Template of *O*-Desmethylangolensin

**M62** possessed the [M − H]^−^ ion at *m*/*z* 257.0817 (C_15_H_13_O_4_, 3.48 ppm) with the retention time at 9.60 min. It was 4 Da more than that of daidzein, which indicated that it might be a dihydrogenation product of daidzein. In its ESI-MS^2^ spectrum, DPIs at *m*/*z* 163 ([M − H − C_6_H_6_O]^−^, produced by RDA rearrangement occurred on positions 3 and 1′) and *m*/*z* 109 ([M − H − C_9_H_8_O_2_]^−^, formed by RDA rearrangement occurred on positions 4 and 10) indicated it should be *O*-desmethylangolensin.

**M63**, eluted at 9.63 min, gave rise to its [M − H]^−^ ion at *m*/*z* 287.0922 (C_16_H_15_O_5_, 2.86 ppm). In the MS^2^, the loss of CH_3_ formed the characteristic ion at *m*/*z* 272. Then the MS^3^ of *m*/*z* 272 presented the specific fragment ion at *m*/*z* 151 via RDA rearrangement occurred on positions 1 and 3. According to the possible metabolic path, the methyl group was attached to 4′-OH to get the hydroxylation and methylation product of **M62**.

### 2.5. Proposed Metabolic Pathways of Genistin−

**Figure 7:**
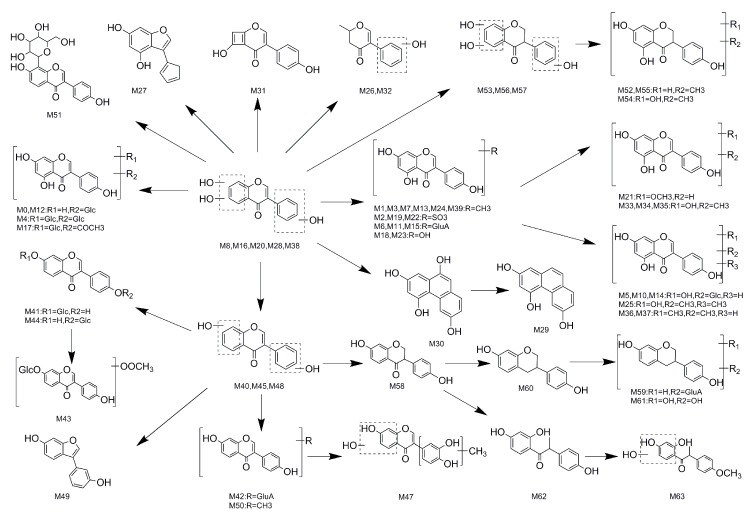
The proposed genistin metabolic patterns *in vivo*.

In our study, a total of 64 metabolites (parent drug included) were found in rats after oral administration of genistin. The proposed metabolic pathways of genistin are illustrated in Figure 7. There was a series of metabolic reactions including methylation, hydrogenation, hydroxylation, glucosylation, glucuronidation, sulfonation, acetylation, ring cleavage and their composite reactions *in vivo* biotransformation. In addition, it should be noted that some cracked ring and rearrangement products were produced, such as **M26**, **M27**, **M29**–**M32**, and **M49**. Furthermore some special products were detected which some methyl groups were introduced to carbon rings, such as **M3** and **M7**.

## 3. Materials and Methods

### 3.1. Chemicals and Reagents

Genistin, genistein, daidzin, daidzein, and puerain were commercially provided by Chengdu Must Biotechnology Co., Ltd. (Chengdu, Sichuan, China). These five reference standards with the purity higher than 98% were applicable to UHPLC-LTQ-Orbitrap analysis. Acetonitrile, methanol, and formic acid (HPLC grade) used in the mobile phase were obtained from Fisher Scientific (Fair Lawn, NJ, USA). In addition, the other reagents and solvents met the requirements of analytical experiments, which were from Beijing Chemical Works (Beijing, China). The ultrapure water was derived from Milli-Q Gradient Å 10 water purification system (Millipore, Billerica, MA, USA). Grace Pure^TM^ SPE C18-Low solid-phase extraction (SPE) cartridges (200 mg/3mL, 59 μm, 70 Å) for solid phase pretreatment of biological samples were supplied by Grace Davison Discovery Science™ (Deerfield, IL, USA).

### 3.2. Animals and Drug Administration

Eight SD rats (male, 200–240 g) were purchased from Beijing Weitong Lihua Experimental Animals Company (Beijing, China) and kept under controlled environmental conditions (temperature: 22–26 °C, relative humidity: 65–75%, day and night alternation time: 12-h light/dark cycle) with free food intake and water consumption. After one week of acclimatization, rats were randomly divided into two groups (*n* = 4 each): Drug Group and Control Group. The standard of genistin was suspended in 0.5% sodium carboxymethyl cellulose (CMC-Na) solution. The Drug Group was orally administered genistin (350 mg/kg), while Control Group was given equivalent 0.5% CMC-Na solution by oral gavage. Before the experiment, all the rats were fasted for 12 h but free drinking water. All procedures were conducted according to the guidelines of Animal Care and Use Committee of Beijing University of Chinese Medicine and Guide for the Care and Use of Laboratory Animals of the US National Institutes of Health.

### 3.3. Sample Collection and Preparation

#### 3.3.1. Plasma Sample Collection

After oral administration, the blood samples were taken from the suborbital venous plexus of the rats in Drug Group at 0.5, 1, 1.5, 2, and 4 h with blood volume of about 0.5 mL. The obtained blood samples were placed in the anticoagulant EP tubes of heparin sodium. After resting for 10 min, each blood sample was centrifuged for 15 min (3500 rpm, 4 °C) and all the blood supernatants in Drug Group were merged into a collective one to get the test plasma containing the drug. The blank plasma sample was from Control Group and the method of collection was the same as the test samples. The above plasma samples were stored at −80 °C.

#### 3.3.2. Urine Sample Collection

Urine samples from 0 to 24 h of each rat in Drug Group were collected by using separate metabolic cages after administration. Each urine sample was centrifuged for 15 min (3500 rpm, 4 °C) and all the urine supernatants in Drug Group were mixed to obtain urine test sample. The blank urine was from Control Group and the method of collection was the same as the test samples. The above urine samples were stored at −80 °C. 

Finally we used the SPE method to pretreat all biological samples, which was a method for precipitation and concentration of protein and solid residues. The SPE cartridges were pre-activated with 5 mL of methanol and 5 mL of deionized water and then 1 mL of plasma and urine samples were added. At last the cartridges were eluted by 5 mL of deionized water and 3 mL of methanol orderly. The methanol eluent was collected and dried under N_2_ at room temperature. The residue was then redissolved in 80 μL 10% acetonitrile solution and centrifuged for 30 min (13,500 rpm, 4 °C). The supernatant was used for subsequent analysis.

### 3.4. Instruments and Analytical Conditions

An UHPLC-LTQ-Orbitrap mass spectrometer (Thermo Scientific, Bremen, Germany) coupled with an ESI source was used to identify metabolites. The separation of samples was performed on a Waters ACQUITY BEH C18 column (2.1 mm × 100 mm i.d., 1.7 μm; Waters Corporation, Milford, MA, USA). The mobile phase consisted of 0.1% formic acid aqueous solution (A) and acetonitrile (B), and the linear gradient procedure was described as follows: 0–2 min, 5–20% B; 2–27 min, 20–85% B; 27–30 min, 85% B. The column temperature was set at 30 °C, and the flow rate was 0.3 mL/min with 2 μL of the injection volume.

The optimized operating parameters were set as follows: capillary temperature, 350 °C; electrospray voltage, 3.5 kV; sheath gas, 35 arb; auxiliary gas, 10 arb; and probe heater temperature, 300 °C. The metabolites were detected by full-scan mass analysis from *m*/*z* 100–1000 with a resolution of 30,000 under positive ion and negative ion modes. The MS^2^ and MS^3^ analysis were based on the data dependent scan, and the three most abundant ions from the precursor list were selected for collision induced dissociation (CID). The collision energy was adjusted to 40% of maximum to minimize the total analysis time. The dynamic exclusion (DE) was used to prevent duplication. The repeat count was set at 5 and the dynamic repeat time was 30 s with the dynamic exclusion duration at 60 s. Furthermore, MS^n^ stages of the obtained data sets were obtained by the parent ion list (PIL)-DE dependent acquisition mode.

### 3.5. Data Processing

The collected data sets were recorded and processed by Thermo Xcalibur 2.1 workstation (Thermo Scientific, Bremen, Germany). In order to acquire as many fragment ions as possible, we selected the peaks with intensity over 10,000 under negative ion mode and 40,000 under positive ion mode to identify the metabolites. Based on the accurate mass and considering the potential element compositions and the occurrence of possible reactions, the types and number of the predicted atoms were set as follows: C [0–35], H [0–50], O [0–25], S [0–2], N [0–3], and ring double bond (RDB) equivalent value [0–15]. The maximum mass errors between the measured and the calculated values were fixed within 5 ppm.

## 4. Conclusions

In the present study, a single oral administration of genistin to SD rats was used to study the metabolic profile of genistin in urine and plasma both in positive and negative ion modes. Different from the previous researches, the multiple metabolite templates were set up to assist in data processing combining with DPIs and calculated Clog *P* values. First, based on the ten metabolite templates, 101 metabolites including the prototype drug were found and confirmed positively or ambiguously according to the fragmentation patterns, accurate mass measurements, chromatographic retention times and relevant drug biotransformation knowledge. Among them, 47 metabolites were found according to the templates of genistin and genistein, which was the same as the previous research method. After using the other metabolite templates, many more metabolites were added, among which 24 metabolites were detected with the templates of daidzin and daidzein. Taking puerarin as a template, only one metabolite was found *in vivo*. There were also six metabolites on the basis of the template of dihydrodaidzein. Moreover, 16 metabolites with the template of hydrogenated genistein (including dihydrogenistein and tetrahydrogenistein), three metabolites based on the template of equol and four metabolites with *O*-desmethylangolensin as the template were identified respectively. After deleting the same metabolites, a total of 64 metabolites (including the parent drug) were left. The metabolite templates and the number of corresponding metabolites were as follows: genistin and genistein (40 metabolites), daidzin and daidzein (11 metabolites), puerarin (one metabolite), dihydrodaidzein (one metabolite), hydrogenated genistein (six metabolites), equol (three metabolites), and *O*-desmethylangolensin (two metabolites). The results demonstrated that genistin mainly underwent methylation, hydrogenation, hydroxylation, glucuronide conjugation, sulfate conjugation, acetylation, ring-cleavage and their composite reactions *in vivo* biotransformation. In conclusion, our research not only revealed the metabolites of genistein *in vivo* roundly, but also proposed an integrated strategy based on multiple metabolite templates to screen and identify the metabolites of natural compounds roundly. Meanwhile, an effective method based on the multiple metabolite templates combined with UHPLC-LTQ-Orbitrap mass spectrometer was established for screening metabolites.

## Figures and Tables

**Table 1:** Summary of genistin metabolites in rat urine and plasma by HPLC-LTQ-Orb.

Peak	Ion Mode	*t*_R_/min	Formula	Theoretical Mass *m*/*z*	Experimental Mass *m*/*z*	Error (ppm)	MS/MS Fragment Ions	Identification/Reactions	Clog *P* Value	U	P
**M0**	P	4.64	C_21_ H_21_O_10_	433.1129	433.1119	−2.34	MS^2^[433]:271(100) MS^3^[271]:153(100), 215(81), 243(66), 271(34), 253(28)	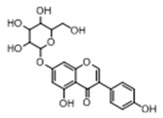	0.91	+	−
	N	4.64	C_21_H_19_O_10_	431.0972	431.0985	2.73	MS^2^[431]:268(100), 269(75) MS^3^[268]:267(100), 224(84), 223(62), 240(54), 239(42), 226(25)			+	−
**M1**	P	3.93	C_16_H_13_O_5_	285.0757	285.0752	−1.93	MS^2^[285]:270(100), 229(18), 225(14), 285(11), 170(11), 145(11) MS^3^[270]:242(100), 152(16), 214(12)	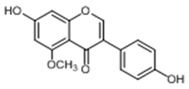	2.09	+	−
	N	3.93	C_16_H_11_O_5_	283.0600	283.0611	3.43	MS^2^[283]:268(100) MS^3^[268]:240(100)			+	−
**M2**	N	3.96	C_15_ H_9_ O_8_ S	349.0012	349.0022	2.77	MS^2^[349]:269(100)	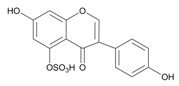	−0.06	+	−
**M3**	P	4.27	C_16_H_13_O_5_	285.0757	285.0753	−1.51	MS^2^[285]:179(100), 165(21) MS^3^[179]:151(100), 109(31), 111(30)	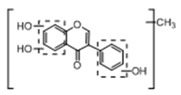	-	+	−
**M4**	P	4.38	C_27_H_31_O_15_	595.1657	595.1645	−2.11	MS^2^[595]:271(100), 433(62), 497(20) MS^3^[271]:243(100), 215(97), 153(39)	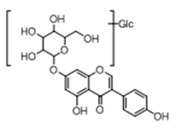	-	+	−
	N	4.38	C_27_H_29_O_15_	593.1501	593.1513	2.05	MS^2^[593]:269(100), 431(14)			+	−
**M5**	P	4.47	C_21_H_21_O_11_	449.1078	449.1065	−1.36	MS^2^[449]:287(100), 272(45), 271(32), 433(14),273(13) MS^3^[287]:187(100), 153(24), 259(3)	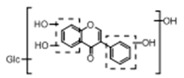	-	−	+
	N	4.47	C_21_H_19_O_11_	447.0922	447.0927	1.17	MS^2^[447]:285(100) MS^3^[285]:241(100), 243(98), 217(85), 175(71), 199(54), 285(50), 201(40), 151(19), 133(18)			−	+
**M6**	P	4.56	C_21_H_19_O_11_	447.0922	447.0910	−2.59	MS^2^[447]:271(100) MS^3^[271]:153(100), 215(98), 243(75), 253(51), 149(38), 159(28)	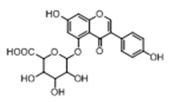	−0.47	+	+
	N	4.56	C_21_H_17_O_11_	445.0765	445.0779	2.95	MS^2^[445]:269(100), 175(26) MS^3^[269]:269(100), 224(37), 241(27), 197(25), 225(21), 181(17), 227(16), 213(14)			+	+
**M7**	P	4.59	C_16_H_13_O_5_	285.0757	285.0752	−2.07	MS^2^[285]:165(100), 270(33), 191(18), 164(11) MS^3^[165]:123(100), 137(66), 109(23), 141(20)	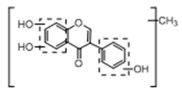	-	+	−
**M8**	P	4.61	C_15_H_11_O_5_	271.0601	271.0595	−2.18	MS^2^[271]:153(100), 215(78), 243(67), 253(37), 149(29), 159(18), 145(18), 271(17), 225(11)	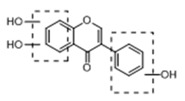	-	+	+
	N	4.61	C_15_H_9_O_5_	269.0444	269.0453	3.23	MS^2^[269]:269(100), 181(57), 201(54), 225(52), 224(35), 197(29), 180(21)			+	+
**M9**	N	4.64	C_15_H_9_O_9_S	364.9961	364.9971	2.44	MS^2^[365]:285(100) MS^3^[285]:257(100), 176(56), 177(52), 243(37), 241(30), 229(26), 217(24)	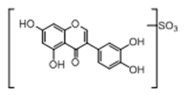	-	+	−
**M10**	N	4.85	C_21_H_19_O_11_	447.0922	447.0927	1.03	MS^2^[447]:284(100), 285(78), 255(15), 270(14), 327(12)	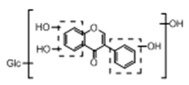	-	−	+
**M11**	P	4.88	C_21_H_19_O_11_	447.0922	447.0905	−3.89	MS^2^[447]:271(100) MS^3^[271]:153(100), 215(95), 243(47), 149(32), 253(31), 145(19)	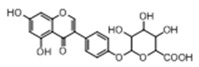	0.43	+	−
	N	4.88	C_21_H_17_O_11_	445.0765	445.0780	3.29	MS^2^[445]:225(100), 201(63), 269(47), 181(38), 213(27), 197(27)			+	+
**M12**	N	5.01	C_21_H_19_O_10_	431.0972	431.0988	3.43	MS^2^[431]:268(100), 269(30) MS^3^[268]:223(100), 224(98), 267(75), 239(47), 240(41), 198(35)	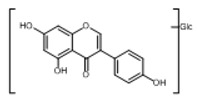	-	+	−
**M13**	N	5.02	C_16_H_11_O_5_	283.0600	283.0609	2.97	MS^2^[283]:268(100) MS^3^[268]:240(100)	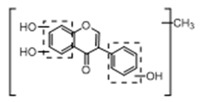	-	+	−
**M14**	N	5.11	C_21_H_19_O_11_	447.0922	447.0937	3.29	MS^2^[447]:271(100), 285(21), 175(16), 403(10)	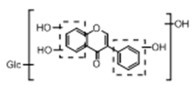	-	+	−
**M15**	N	5.11	C_21_H_17_O_11_	445.0765	445.0780	3.29	MS^2^[445]:269(100), 175(15), 153(12) MS^3^[269]:225 (100), 201(55), 241(28), 197(24), 269(18)	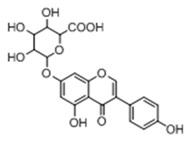	0.43	+	−
**M16**	P	5.52	C_15_H_11_O_5_	271.0601	271.0596	−1.84	MS^2^[271]:215(100), 153(82), 243(61), 149(38), 253(36), 159(20), 145(14), 225(13)	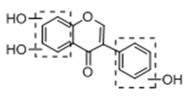	-	+	−
**M17**	N	5.52	C_23_H_21_O_11_	473.1079	473.1092	2.80	MS^2^[473]:268(100), 269(66), 311(12)	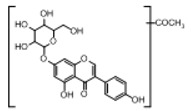	-	+	−
**M18**	N	5.82	C_15_H_9_O_6_	285.0393	285.0400	2.37	MS^2^[285]:217(100), 199(19), 241(16), 175(10)	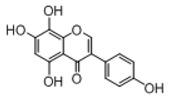	1.77	+	−
**M19**	P	5.83	C_15_H_11_O_8_S	351.0169	351.0162	−1.92	MS^2^[351]:271(100), 333(21) MS^3^[271]:153(100), 253(81), 215(63), 243(50), 159(32), 149(22), 197(12)	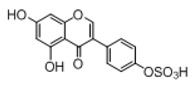	0.84	+	−
	N	5.83	C_15_H_9_O_8_S	349.0012	349.0023	2.94	MS^2^[349]:269(100) MS^3^[269]:225(100), 227(95), 175(51), 269(36), 241(32), 240(30), 197(24), 224(23)			+	−
**M20**	N	5.92	C_15_H_9_O_5_	269.0444	269.0454	3.68	MS^2^[269]:225(100), 269(96), 181(71), 227(68), 201(54), 224(43), 197(41)	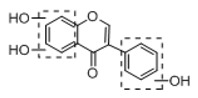	-	+	−
**M21**	N	5.92	C_16_H_11_O_6_	299.0549	299.0559	2.89	MS^2^[299]:269(100) MS^3^[269]:241(100), 224(48), 173(43), 180(22)	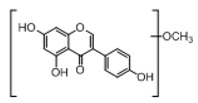	-	+	−
**M22**	N	6.38	C_15_H_9_O_8_S	349.0012	349.0018	1.45	MS^2^[349]:269(100) MS^3^[269]:225(100), 227(65), 269(39), 176(31), 197(26), 181(22), 169(21), 213(12), 241(10)	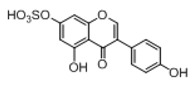	0.85	+	−
**M23**	P	6.53	C_15_H_11_O_6_	287.0550	287.0544	−2.32	MS^2^[287]:272(100), 286(46), 153(44), 241(39), 269(34), 271(33), 231(19), 287(18), 259(13)	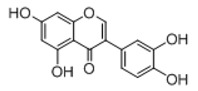	1.81	+	−
**M24**	P	6.62	C_16_H_13_O_5_	285.0757	285.0751	−2.28	MS^2^[285]:285(100), 270(90), 229(17), 225(15) MS^3^[270]:242(100), 152(11)	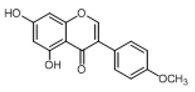	2.99	+	−
	N	6.62	C_16_H_11_O_5_	283.0600	283.0610	3.32	MS^2^[283]:268(100) MS^3^[268]:240(100)			+	−
**M25**	P	6.98	C_17_H_15_O_6_	315.0864	315.0856	−2.36	MS^2^[315]:300(100), 283(20) MS^3^[300]:167(100), 283(69), 299(42), 244(36), 166(32), 272(27)	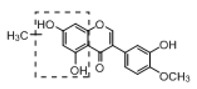	-	+	−
**M26**	N	7.93	C_12_H_9_O_3_	201.0570	201.0553	3.38	MS^2^[201]:173(100), 159(94), 157(75)	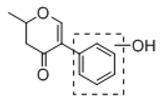	-	+	−
**M27**	P	7.97	C_13_H_11_O_3_	215.0702	215.0697	−2.65	MS^2^[215]:197(100), 169(64), 187(31), 147(18), 173(15), 159(10)	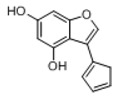	2.67	+	−
**M28**	P	8.03	C_15_H_11_O_5_	271.0601	271.0590	−3.98	MS^2^[271]:271(100), 153(32), 215(25), 243(19) MS^3^[271]:153(100), 215(71), 243(58), 253(35), 149(25)	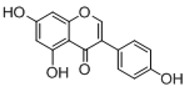	2.41	+	+
	N	8.03	C_15_H_9_O_5_	269.0444	269.0442	−1.08	MS^2^[269]:269(100), 225(52), 181(47), 201(44), 224(32), 197(26), 241(23)			+	+
**M29**	N	8.03	C_14_H_9_O_3_	225.0546	225.0546	−0.09	MS^2^[225]:197(100), 181(98), 196(33), 169(27), 183(19), 180(17)	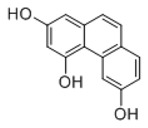	2.49	+	−
**M30**	N	8.03	C_14_H_9_O_4_	241.0495	241.0494	−0.52	MS^2^[241]:213(100), 197(56), 199(22), 169(14), 196(11), 173(11)	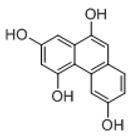	1.82	+	−
**M31**	N	8.03	C_13_H_7_O_4_	227.0340	227.0339	−0.07	MS^2^[227]:183(100), 199(40), 155(19), 66(13), 159(10)	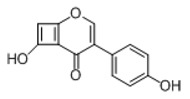	0.49	+	−
**M32**	N	8.14	C_12_H_9_O_3_	201.0570	201.0552	2.78	MS^2^[201]:159(100), 173(98), 133(18)	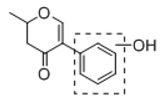	-	+	−
**M33**	P	8.24	C_16_ H_13_O_6_	301.0706	301.0700	−2.17	MS^2^[301]:286(100) MS^3^[286]:285(100), 258(31), 286(14), 168(13)	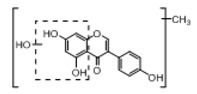	-	+	−
**M34**	P	8.46	C_16_H_13_O_6_	301.0706	301.0700	−2.37	MS^2^[301]:286(100), 269(41), 241(14) MS^3^[286]:258(100), 153(83), 285(21), 269(12)	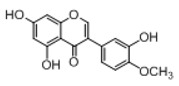	2.25	+	−
	N	8.46	C_16_H_11_O_6_	299.0549	299.0559	2.89	MS^2^[299]:284(100) MS^3^[284]:256(100), 255(60), 227(37), 283(30), 212(30), 211(24), 240(20), 239(15)			+	−
**M35**	P	8.79	C_16_H_13_O_6_	301.0706	301.0700	−2.08	MS^2^[301]:286(100), 269(74), 241(25), 153(17) MS^3^[286]:153(100), 258(36), 229(21), 269(14), 230(10)	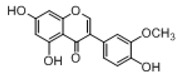	2.25	+	−
**M36**	P	10.07	C_17_H_15_O_5_	299.0903	299.0906	−2.84	MS^2^[299]:284(100) MS^3^[284]:256(100), 253(53), 161(32), 269(32), 108(27), 228(27), 152(21)	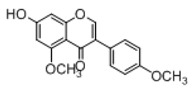	2.67	+	−
**M37**	P	10.28	C_17_H_15_O_5_	299.0903	299.0905	−3.08	MS^2^[299]:284(100), 243(20), 166(20), 299(13), 239(12), 267(11) MS^3^[284]:256(100), 166(25), 267(11), 255(11), 283(10)	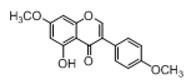	3.57	+	−
**M38**	N	12.34	C_15_H_9_O_5_	269.0444	269.0453	3.23	MS^2^[269]:269(100), 181(61), 201(55), 225(53), 224(40), 197(33), 180(26), 183(23), 133(11)	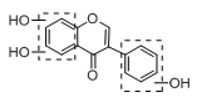	-	+	−
**M39**	P	12.44	C_16_H_13_O_5_	285.0757	285.0749	−3.02	MS^2^[285]:270(100), 229(49), 253(42), 152(31), 123(25), 269(19) MS^3^[270]:152(100), 269(87), 242(40), 253(36), 252(31)	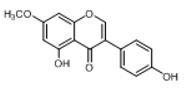	2.99	+	−
	N	12.44	C_16_H_11_O_5_	283.0600	283.0609	2.97	MS^2^[283]:268(100) MS^3^[268]:267(100), 239(68), 224(68), 240(61), 223(43), 226(22), 211(11)			+	−
**M40**	P	3.85	C_15_H_11_O_4_	255.0652	255.0647	−1.90	MS^2^[255]:199(100), 137(65), 227(52), 237(23), 145(12)	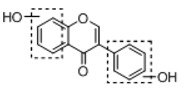	-	+	+
	N	3.85	C_15_H_11_O_4_	253.0495	253.0505	3.77	MS^2^[253]:253(100), 224(52), 209(43), 225(35), 197(26), 208(18), 124(14), 223(11)			+	−
**M41**	P	3.86	C_21_H_21_O_9_	417.1180	417.1170	−2.42	MS^2^[417]:255(100) MS^3^[255]:199(100), 227(45), 181(40), 137(24), 209(20)	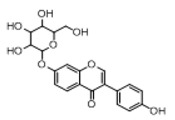	0.37	+	−
	N	3.86	C_21_H_19_O_9_	415.1023	415.1035	2.73	MS^2^[415]:253(100), 252(15) MS^3^[253]:253(100), 135(53), 160(46), 225(27)			+	−
**M42**	P	3.86	C_21_H_19_O_10_	431.0973	431.0960	−2.44	MS^2^[431]:255(100) MS^3^[255]:199(100), 137(33), 255(28), 227(19), 145(16), 211(10)	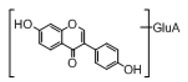	-	+	−
	N	3.86	C_21_H_17_O_10_	429.0815	429.0830	3.16	MS^2^[429]:253(100), 175(41) MS^3^[253]:209(100), 253(91), 183(23)			+	−
**M43**	N	3.86	C_22_H_21_O_11_	461.1078	461.1091	2.80	MS^2^[461]:253(100), 415(37), 298(15), 240(13), 284(12)	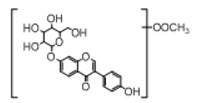	-	+	−
**M44**	P	4.26	C_21_H_21_O_9_	417.1180	417.1164	−3.81	MS^2^[417]:255(100) MS^3^[255]:199(100), 237(53), 137(47), 227(22), 157(10)	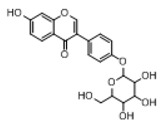	0.56	+	−
	N	4.26	C_21_H_19_O_9_	415.1023	415.1035	2.73	MS^2^[415]:253(100), 135(53), 225(27)			+	−
**M45**	N	4.82	C_15_H_9_O_4_	253.0495	253.0505	3.62	MS^2^[253]:253(100), 224(52), 225(39), 209(33), 197(25), 208(12)	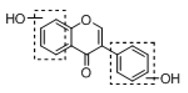	-	+	−
**M46**	P	4.88	C_15_H_11_O_7_S	335.0220	335.0208	−3.55	MS^2^[335]:255(100), 316(44) MS^3^[255]:199(100),137(33),255(28), 227(19), 145(16), 211(10)	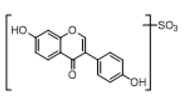	-	+	−
	N	4.88	C_15_H_9_O_7_S	333.0063	333.0071	0.79	MS^2^[333]:253(100) MS^3^[253]:211(100), 209(95), 225(91), 224(56), 135(42)			+	−
**M47**	N	6.31	C_16_H_11_O_6_	299.0549	299.0549	−0.48	MS^2^[299]:281(100), 163(79), 253(39), 237(28), 225(13), 224(11)	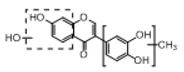	-	+	−
**M48**	P	6.33	C_15_H_11_O_4_	255.0652	255.0648	−1.67	MS^2^[255]:255(100), 199(18), 137(13) MS^3^[255]:199(100), 137(97), 227(42), 171(12)	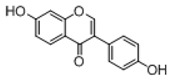	2.08	+	+
	N	6.33	C_15_H_9_O_4_	253.0495	253.0499	1.56	MS^2^[253]:253(100) MS^3^[253]:253(100), 225(53), 209(46), 197(18), 208(17)			+	+
**M49**	P	6.34	C_14_H_11_O_3_	227.0703	227.0698	−2.16	MS^2^[227]:182(100), 199(47), 184(26), 157(22), 86(22)	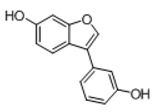	3.26	+	−
**M50**	P	9.92	C_16_H_13_O_4_	269.0803	269.0801	−2.58	MS^2^[269]:254(100), 237(46), 213(37), 253(12)	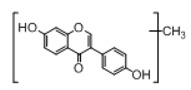	-	+	−
**M51**	N	3.49	C_21_H_19_O_9_	415.1023	415.1036	2.94	MS^2^[415]:295(100) MS^3^[295]:267(100)	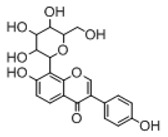	0.02	+	−
**M52**	P	4.16	C_16_H_15_O_5_	287.0915	287.0908	−2.23	MS^2^[287]:259(100), 66(30), 231(18), 241(18), 213(14)	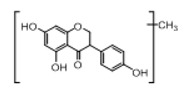	-	+	−
**M53**	P	5.13	C_15_H_13_O_5_	273.0758	273.0751	−2.27	MS^2^[273]:255(100), 66(85), 179(82), 123(58), 245(31)	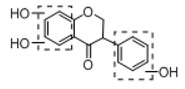	-	+	−
**M54**	P	5.19	C_16_H_15_O_6_	303.0863	303.0870	2.39	MS^2^[303]:230(100), 285(87), 217(30), 257(22), 272(14)	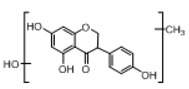	-	+	−
**M55**	P	6.72	C_16_H_15_O_5_	287.0915	287.0906	−2.75	MS^2^[287]:272(100), 286(40), 66(38), 270(36), 193(29)	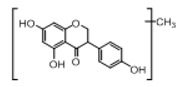	-	+	−
**M56**	P	7.64	C_15_H_13_O_5_	273.0758	273.0751	−2.49	MS^2^[273]:255(100), 179(84), 123(42), 151(37), 107(10), 245(10)	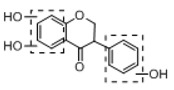	-	+	−
	N	7.64	C_15_H_11_O_5_	271.0601	271.0609	3.10	MS^2^[271]: 165 (100), MS^3^[165]:137(100), 93(26), 109(25), 121(24)			+	−
**M57**	P	8.03	C_15_H_13_O_5_	273.0758	273.0750	−2.86	MS^2^[273]:272(100), 216(49), 244(41), 153(41), 154(37), 254(24)	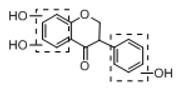	-	+	−
**M58**	P	6.42	C_15_H_13_O_4_	257.0809	257.0802	−2.47	MS^2^[257]:140(100), 256(76), 163(53), 200(30), 137(29) MS^3^[140]:112(100)	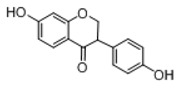	2.53	+	−
	N	6.37	C_15_H_11_O_4_	255.0652	255.0649	−1.32	MS^2^[255]:149(100), 254(79), 135(20) MS^3^[149]:121(100)			+	−
**M59**	N	5.20	C_21_H_21_O_9_	417.1181	417.1194	3.38	MS^2^[417]:175(100), 399(25), 113(21), 227(12), 109(11)	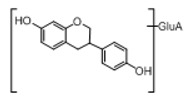	-	+	+
**M60**	P	5.23	C_15_H_15_O_3_	287.0915	243.1008	−3.05	MS^2^[243]:172(100), 200(63), 198(56), 197(33), 216(33), 225(18), 123(10) MS^3^[172]:157(100)	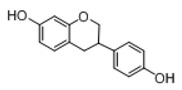	3.01	+	−
**M61**	P	6.33	C_15_H_15_O_5_	275.0908	275.0911	−1.24	MS^2^[275]:151(100) MS^3^[151]:123(100), 151(71), 133(48), 141(33), 107(17)	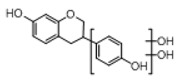	-	+	−
**M62**	N	9.60	C_15_H_13_O_4_	257.0809	257.0817	3.48	MS^2^[257]:239(100), 109(69), 163(36), 213(30), 242(27), 148(20)	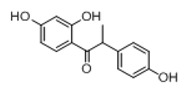	2.82	+	−
**M63**	P	9.63	C_16_H_17_O_5_	289.1065	289.1061	−3.36	MS^2^[289]:149(100), 271(76), 121(26), 195(20), 66(19) MS^3^[149]:121(100)	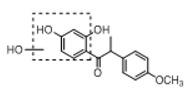	-	+	−
	N	9.63	C_16_H_15_O_5_	287.0913	287.0922	2.86	MS^2^[287]:272(100) MS^3^[272]:152(100), 151(34)			+	−

Note: *t*_R_: retention time; U: urine; P: plasma; +: detected; −: undetected.
